# Cystic Artery: Morphological Study and Surgical Significance

**DOI:** 10.1155/2016/7201858

**Published:** 2016-10-16

**Authors:** Usha Dandekar, Kundankumar Dandekar

**Affiliations:** ^1^Department of Anatomy, SMBT Institute of Medical Sciences and Research Centre, Dhamangaon, Nasik District, Maharashtra 422403, India; ^2^Department of General Surgery, SMBT Institute of Medical Sciences and Research Centre, Dhamangaon, Nasik District, Maharashtra 422403, India

## Abstract

The cystic artery is the key structure sought to be clipped or ligated during laparoscopic or conventional cholecystectomy. The possible complications like hemorrhage or hepatobiliary injury are always centered on the search, dissection, and clipping or ligation of the cystic artery, many a time because of possibility of variations in its course and relations to the biliary ducts. This descriptive study was carried out to document the normal anatomy and different variations of the cystic artery to contribute to improve surgical safety. This study conducted on 82 cadavers revealed cystic artery with mean length of 16.9 mm (ranged between 2 mm and 55 mm) and mean diameter of 1.6 mm (range between 1 mm and 5 mm). The origin of cystic artery from celiac right hepatic artery was found in 79.3% and in the remaining 20.7% it was replaced. Single cystic artery was present in 72% and double cystic artery in 28%. Considering the site of origin of the cystic artery with reference to Calot's triangle, it was observed within the triangle in 62.2% and outside it in 37.8%. All the cystic arteries passed through Calot's triangle except for 3.6%. The cystic artery crossed the common hepatic duct anteriorly in 26.8% and posteriorly in 6.1%. It crossed common bile duct anteriorly in 1.2% and posteriorly in 3.7%. The knowledge of such variations and its awareness will decrease morbidity and help to keep away from a number of surgical complications during cholecystectomy.

## 1. Introduction

The importance of the cystic artery (CA) goes hand in hand with the history of cholecystectomy. By 1890, the early modern surgeons were having doubts over the utility of cholecystectomy but gradually cholecystectomy was accepted. But even after acceptance and wide application, the aura of apprehension remained with cholecystectomy because of recurring complications. The CA always remained the center of attraction as complications were centered on key step of ligating and dividing the CA. Today laparoscopic cholecystectomy is widely performed all over the world. In India it is an established procedure in urban centers and rapidly spreading to peripheral centers. The CA is the key structure sought to be clipped or ligated during laparoscopic or conventional cholecystectomy. The possible complications like hemorrhage or hepatobiliary injury are always centered on the search, dissection, and clipping or ligation of CA. The common reason is the possibility of variation in the course of CA and its relations to the biliary ducts. All these recurring complications are a cause of attraction for surgeons, radiologists, and anatomists to study this artery persistently.

The CA usually arises from the right hepatic artery (RHA) to the right of common hepatic duct (CHD) in Calot's triangle. On reaching the gall bladder neck, it divides into superficial and deep branches to supply free peritoneal surface and attached nonperitoneal surface of the gall bladder (GB), respectively. The branches anastomose over the surface of body and fundus of the gall bladder and give off numerous twigs to the liver substance [1, 2]. In 25% of subjects, the superficial and deep branches of the CA have separate origins and Michels called them double CA [3]. The CA is always mentioned in relation to Calot's triangle which was first described by Calot in 1891 as bounded by the cystic duct (CD), CHD, and the CA. In 1981, Rocko et al. drew attention to possible variations in the region of Calot's triangle and defined a triangle bordered by the CD, CHD, and lower edge of the liver [4]. In 1992, Hugh et al. suggested Calot's triangle should be renamed as the hepatobiliary triangle [5]. Anatomic variations in and around Calot's triangle are frequent. Therefore, careful dissection of Calot's triangle is necessary for both conventional and laparoscopic cholecystectomy. Hemorrhage could be a problem during search of the CA if these variations are overlooked and that increases the rate of conversion to open surgery. It also needs to be kept in mind that, during laparoscopic visualization, anatomical relations are seen differently compared to during conventional cholecystectomy [6]. This emphasizes the importance of cystic arterial dissection and necessity of thorough knowledge of cystic arterial variations for safe performance of cholecystectomy. The aim of this cross-sectional, observational, quantitative, and descriptive study is to record the normal and variant anatomy of CA in the hope of providing contribution towards increasing the safety of cholecystectomy.

## 2. Material and Methods

Eighty-two adult formalin embalmed cadavers (males: 72, females: 10) representing a heterogeneous sample from local population were used. Cadavers with any operative procedure and with presence of any pathology in subhepatic region were excluded. The subhepatic region of the abdomen was exposed by separating the lesser omentum with gross dissection followed by fine dissection to display the CA. The origin, number, length, and course of the CA along with its position with reference to Calot's triangle were recorded. In addition to this, its relations to biliary ducts were also noted. Findings of every cadaveric dissection were meticulously recorded and extensively photographed. Finally, all collected data was analyzed and expressed as percentage.

## 3. Results

**Figure 1:**
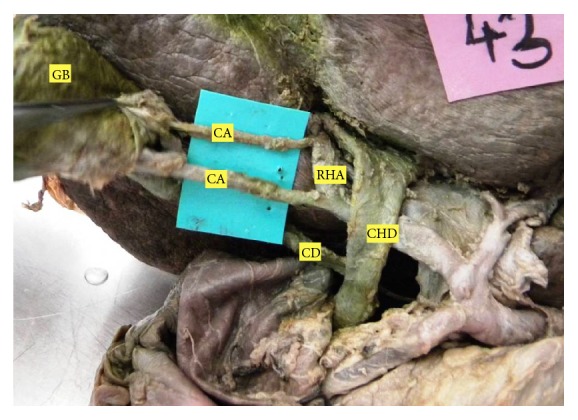
Double CA; both superficial and deep branches arising from RHA.

The CA originated from celiac RHA in 79.3% and in the remaining 20.7% it was replaced, where it originated from aberrant right hepatic artery (ARHA) in 12.1%, from hepatic artery proper (HAP) in 3.7%, from common hepatic artery (CHA) in 2.5%, from middle hepatic artery (MHA) in 1.2%, and from left hepatic artery (LHA) in 1.2%. When we consider the origin of CA from RHA including ARHA, it becomes 91.4%. The mean length of the CA was 16.9 mm and ranged between 2 mm and 55 mm. The mean diameter of the CA was 1.6 mm and ranged between 1 mm and 5 mm. The CA dividing into superficial and deep branches was seen in 72% and in the remaining 28% it did not divide but instead supplied only superficial surface of gall bladder. In the later cases, the deep branch of double CA had separate origin from RHA (18.3%), ARHA (7.3%), HAP (1.2%), or gastroduodenal artery (GDA) (1.2%). The superficial branch of double CA arose from RHA (21.9%), ARHA (2.4%), HAP (1.2%), LHA (1.2%), or CHA (1.2%) (Figure 1).

**Figure 2:**
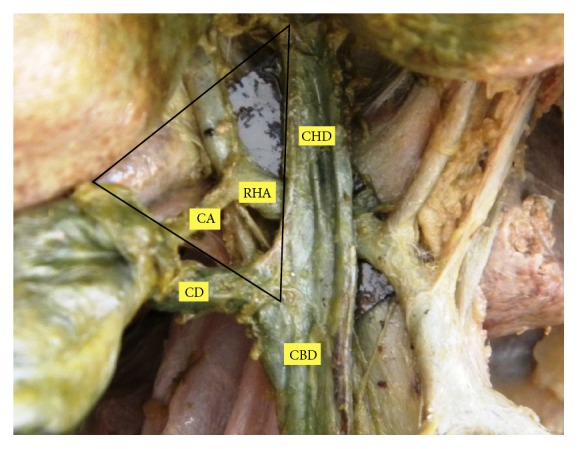
CA arising from RHA within Calot's triangle.

**Figure 3:**
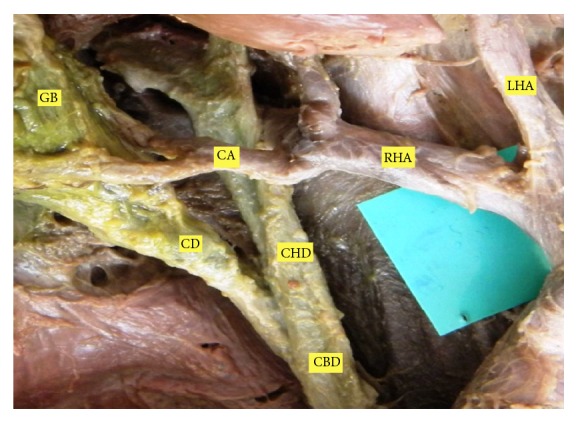
CA originating outside Calot's triangle and coursing anterior to CHD.

**Figure 4:**
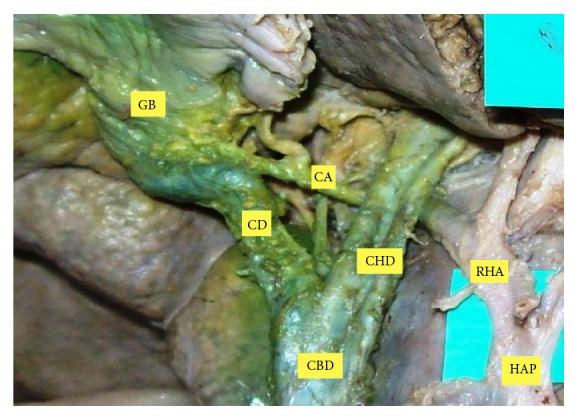
CA coursing posterior to CHD.

The position of CA was variable in Calot's triangle. The CA passing through middle portion of Calot's triangle was seen to be in higher proportion (Table 1). In 3 cases (3.6%), it did not enter in the Calot's triangle. The incidence of the CA originating within Calot's triangle was found in 62.2% (Figure 2). Out of these incidences, it arose from RHA in 52.5% and from ARHA in 9.7% of cases. The CA originated outside Calot's triangle in 37.8%, where it passed anterior to the CHD in 26.8% (Figure 3) and posterior to it in 6.1% (Figure 4). In relation to CBD, the CA was anteriorly placed in 1.2% and posterior to it in 3.7% of cases.

## 4. Discussion

Many years ago, Sir Arthur Keith stressed the fact that, in the biliary region, “variation is rampant.” This phrase, altered to read “variation is constant,” is aptly applicable to the blood supply of the supramesocolic organs [3]. Michels [20] quoted that, according to Lahey, “cholecystectomy is a dangerous operation unless one realizes that variations are very common.” The CA varies in number, origin, course, and its relations to biliary ducts. These abnormalities have been mentioned from time to time in literature [3, 5, 7–19, 21–24].

The CA is usually a branch of celiac RHA. It may arise from the LHA, CHA, HAP, ARHA, GDA, superior mesenteric artery (SMA), celiac trunk, or aorta [2]. In the present study, we found the origin of the CA from RHA in 79.3% and from sources other than RHA in 20.7%. McVay [25] divided the types of origin of CA into 4 categories: Category I: (1) from RHA, (2) from HAP at the point of division, (3) from LHA, and (4) from HAP proximal to the point of division; Category II: from GDA or SPDA; Category III: from the same arteries mentioned in Category I but differing from them in the deviation of the parent vessel; Category IV: from right gastric artery, CHA, celiac trunk, or SMA.

Incidence of variations in the origin of cystic artery is compared with other authors' studies (Table 2). When we consider the origin of CA from RHA including ARHA, it was 91.4%. Michels [3], Pushpalatha and Shamasundar [10], Johnston and Anson [11], Tejaswi et al. [12], Daseler et al. [13], and Gawali [15] found this incidence in 89.5%, 56%, 100%, 96%, 87.8%, and 93.3%, respectively. We found origin of CA from MHA in 1.2%. Such finding was not observed by any other authors except Bhardwaj [16]. Though Michels [3] found origin of CA from LHA and MHA in 5%, the incidence of origin from MHA was not clearly mentioned.

Mean length of the CA was 16.9 mm and ranged between 2 mm and 55 mm. Tejaswi et al. [12] reported mean length of CA to be 17.6 mm with a range of 3.7 mm–42 mm. Taimur et al. [17] divided CA according to its length into three groups: short (less than 1 cm = 7%), normal (1–3 cm = 82%), and long (more than 3 cm = 8%). We found this incidence in 17.1%, 73.2%, and 9.7%, respectively. Surgeons should be aware of such types as short CA may easily be avulsed from the hepatic artery if excessive traction is applied to the gall bladder [26].

The incidence of double CA ranges from 15 to 25% [2]. Such arteries usually arise from RHA and frequently replace the deep branch of the CA. The absence of a deep branch close to the gallbladder may be a clue that doubling of the CA is present [5]. In the present study, we found double CA in 28% which is considerably higher than that reported by other authors (Michels 25% [3], Hugh et al. 22% [5], Saidi et al. 7.8% [7], Johnston and Anson 17.1% [11], Tejaswi et al. 3% [12], Daseler et al. 14% [13], Flint 15.5% [14], Gawali 13.3% [15], Bhardwaj 10% [16], Taimur et al. 6% [17], Ding et al. 13.7% [18], Balija et al. 15.5% [21], Antonetti and Diaz 18.3% [19], Vandamme et al. 16% [22], Sugita et al. 19% [23], and Zubair et al. 18.2% [24]). This indicates that in 1/4th of cases there is a possibility of double CAs suggesting that one should have high index of suspicion for double CAs while operating in this region.

Michels [3] stated that the superficial branch of double CA arose from the RHA, LHA, MHA, GDA, or retroduodenal artery. The deep branch arose from the RHA only. In the present study, the superficial branch arose from RHA, ARHA, HAP, CHA, or LHA and the deep branch arose from RHA, ARHA, HAP, or GDA. The common origin of both superficial and deep branches of double CA from RHA was seen in 18.3% in present study. Michels [3], Johnston and Anson [11], Flint [14], and Antonetti and Diaz [19] found this incidence in 15.5%, 11.4%, 8%, and 7.3%, respectively. Tripling of the CA is very rare and was encountered in only 1 case by Michels [3] and1 case by Daseler et al. [13]. We did not come across such variation during this study.

Regarding the site of origin of CA, it usually arises within Calot's triangle. The comparison of our findings with other authors' observations is shown in Table 3. Out of 62.2 % of CA arising within Calot's triangle, the incidence of origin of CA from celiac RHA was seen in 52.5%. It was noted in 63%, 40%, and 74.8% by other authors [3, 11, 18]. In addition to the variability in origin and position of CA, its course may also follow diverse path, often in close proximity to the CHD [6]. These findings are compared with findings of other authors in Table 4. In the present study, the CA was mostly found to be at a higher level than the commencement of CBD. These findings are correlating with the findings of other authors [11, 15, 19] though few cases were seen to be related to the CBD similar to Saidi et al.'s study [7]. It can be said that during surgery the presence of CA should be sought above the level of commencement of CBD.

The explanation for variant CA is found in the developmental pattern of the biliary system. During fetal development, the gall bladder develops from hepatic diverticulum of the foregut which is richly supplied by abdominal aorta and its initial branches. Later most of these vessels degenerate to form the mature vascular system, because the pattern of degeneration is highly variable, probably resulting in variations of blood supply [27].

### 4.1. Surgical Significance of Cystic Artery

1. The CA originating outside Calot's triangle usually passes anterior to bile ducts and in some cases may remain posterior to the CD; thus, it becomes the first structure encountered during exploration of Calot's triangle having proneness for injury [5].
2. When CA courses anterior to CHD or CBD, the close proximity increases the chances of injury to CHD or CBD [26].
3. An accessory CA is liable to get torn and bleed if not identified [26].
4. A Caterpillar hump RHA can be mistaken for CA and ligated inadvertently leading to morbid complications [28].
5. The CA arising from the “Caterpillar hump RHA” is typically short and may get avulsed from RHA if excessive traction is applied to gall bladder [26].
6. Presence of abnormally large CA may be taken as warning sign for possibility of presence of a “Caterpillar hump RHA” coursing near the CD and gall bladder infundibulum [29].
7. From laparoscopic viewpoint, a single “large cystic artery” requires special attention during exploration, as it may actually be an aberrant hepatic artery, which needs to be carefully isolated from the CD or gallbladder [21].
8. It is safer to clip or ligate superficial and deep branches of CA near the gall bladder to avoid confusion due to fair number of variations of CA [30].
9. In case of double CA, the deep branch is so delicate that, being unrecognized, it is cut during dissection [21].
10. The CA gives off direct branches to the CD. These vessels have to be divided to obtain a length of CD before division [30].

## 5. Conclusion

The cystic artery is a key anatomical structure to be isolated and ligated during laparoscopic or conventional cholecystectomy. The possible hemorrhage or hepatobiliary complications are known to occur during the search, dissection, or ligation/clipping of cystic artery. It is crucial for the surgeon to give careful attention, identify, and confirm the cystic artery before clipping or ligation. Thus, it is essential from the surgeon's viewpoint to have a thorough knowledge and awareness of variations of cystic artery which will contribute to the safety of cholecystectomy.

## Figures and Tables

**Table 1:** Position of cystic artery within Calot's triangle.

Position of CA in Calot's triangle	Number of specimens
Upper part	12.2%
Middle part	47.6%
Lower part	36.6%

**Table 2:** Variation in source of origin of the cystic artery.

Studies	Source of origin								
	RHA	ARHA	HAP	CHA	LHA	MHA	GDA	SMA	CT
Michels [3]	77.5	12	0	1.5	5	0	4	0	0
Saidi et al. [7]	92.2	0	7.8	0	0	0	0	0	0
Bakheit [8]	78	0	0	17	2	0	3	0	0
Khalil et al. [9]	90	0	5	0	3	0	2	0	0
Pushpalatha and Shamasundar [10]	54	2	22	12	0	0	8	2	0
Johnston and Anson [11]	85.7	14.3	0	0	0	0	0	0	0
Tejaswi et al. [12]	92	4	2	0	1	0	1	0	0
Daseler et al. [13]	71.7	16.1	0	2.8	6.3	0	2.6	0.1	0.3
Flint [14]	98	0	0	0	1.5	0	0.5	0	0
Gawali [15]	90	3.3	0	0	3.3	0	3.3	0	0
Bhardwaj [16]	75	0	0	0	5	13.3	6.7	0	0
Present study	79.3	12.1	3.7	2.5	1.2	1.2	0	0	0

**Table 3:** Comparison of site of origin of cystic artery in relation to Calot's triangle with other studies.

Authors	Site of origin of CA	
	Inside Calot's triangle %	Outside Calot's triangle %
Michels [3]	81	19
Saidi et al. [7]	2	98
Bakheit [8]	25	75
Tejaswi et al. [12]	65	35
Daseler et al. [13]	69.8	30.2
Flint [14]	84	16
Gawali [15]	90	10
Taimur et al. [17]	88	9
Ding et al. [18]	87	13
Present study	62.2	37.8

**Table 4:** Variation in relation of cystic artery with CHD and CBD.

Relations to ducts	Authors					
	Saidi et al. [7] %	Bakheit [8] %	Johnston and Anson [11] %	Gawali [15] %	Antonetti and Diaz [19] %	Present study %
Common hepatic duct						
Anterior	45.1	7	31.4	46.7	26.8	26.8
Posterior	46.1	—	2.9	50	9.7	6.1

Common bile duct						
Anterior	2.9	2	—	—	—	1.2
Posterior	3.9	—	—	—	—	3.7

## Abbreviations

CA: Cystic artery
RHA: Right hepatic artery
CD: Cystic duct
GB: Gall bladder
CHD: Common hepatic duct
ARHA: Aberrant right hepatic artery
HAP: Hepatic artery proper
CHA: Common hepatic artery
MHA: Middle hepatic artery
LHA: Left hepatic artery
GDA: Gastroduodenal artery
SMA: Superior mesenteric artery
CBD: Common bile duct.
